# Two novel colorectal cancer risk loci in the region on chromosome 9q22.32

**DOI:** 10.18632/oncotarget.24340

**Published:** 2018-01-29

**Authors:** Jessada Thutkawkorapin, Hovsep Mahdessian, Tom Barber, Simone Picelli, Susanna von Holst, Johanna Lundin, Laura Valle, Vinaykumar Kontham, Tao Liu, Daniel Nilsson, Xiang Jiao, Annika Lindblom

**Affiliations:** ^1^ Department of Molecular Medicine and Surgery, Karolinska Institute, Stockholm SE-17176, Sweden; ^2^ The Ludwig Center and Howard Hughes Medical Institute at the Sidney Kimmel Comprehensive Cancer Center at Johns Hopkins, Baltimore, MD 21231, USA; ^3^ Hereditary Cancer Program, Catalan Institute of Oncology, IDIBELL and CIBERONC, Barcelona 08908, Spain

**Keywords:** familial colorectal cancer, risk haplotype, cancer predisposition, association study, next generation sequencing

## Abstract

Highly penetrant cancer syndromes account for less than 5% of all cases with familial colorectal cancer (CRC), and other genetic contribution explains the majority of the genetic contribution to CRC. A CRC susceptibility locus on chromosome 9q has been suggested. In this study, families where risk of CRC was linked to the region, were used to search for predisposing mutations in all genes in the region. No disease-causing mutation was found. Next, haplotype association studies were performed in the region, comparing Swedish CRC cases (2664) and controls (4782). Two overlapping haplotypes were suggested. One 10-SNP haplotype was indicated in familial CRC (OR 1.4, *p* = 0.00005) and one 25-SNP haplotype was indicated in sporadic CRC (OR 2.2, *p* = 0.0000012). The allele frequencies of the 10-SNP and the 25-SNP haplotypes were 13.7% and 2.5% respectively and both included one RNA, *RP11-332M4.1* and *RP11-l80l4.2*, in the non-overlapping regions. The sporadic 25-SNP haplotype could not be studied further, but the familial 10-SNP haplotype was analyzed in 61 additional CRC families, and 6 of them were informative for all markers and had the risk haplotype. Targeted sequencing of the 10-SNP region in the linked families identified one variant in *RP11-332M4.1*, suggestive to confer the increased CRC risk on this haplotype. Our results support the presence of two loci at 9q22.32, each with one RNA as the putative cause of increased CRC risk. These RNAs could exert their effect through the same, or different, genes/pathways, possibly through the regulation of neighboring genes, such as *PTCH1, FANCC, DKFZP434H0512, ERCC6L2* or the processed transcript *LINC00046*.

## INTRODUCTION

It has been estimated that approximately 13% of all colorectal cancers (CRC) may be due to genetic factors [1]. However, the known predisposing inherited polyposis- and non-polyposis syndromes with highly penetrant mutations in *APC*, *MUTYH*, the DNA mismatch repair (MMR) genes and other even more rare genes, account for less than 5% of all cases [2]. Hypothetically, other high-risk and low-risk genes would explain the majority of the genetic contribution to CRC. Genome-wide linkage analysis (GWL) in CRC families has traditionally been used to identify high-penetrant genes, while genome-wide association studies (GWAS) in CRC patients and controls have been used to find alleles associated with a low/modest risk. Several candidate regions linked to CRC predisposition have been identified by GWL, however, yet no new disease gene or syndrome has been identified in those genomic regions [3, 4]. In the last years, GWAS have identified alleles associated with a small increased CRC risk, altogether considered to contribute to a minor part of the missing heritability in CRC [5]*.* At the same time, whole-exome sequencing studies in CRC families have revealed novel rare candidate genes for hereditary CRC, with more or less evidence of causality [6–11].

A sib pair study identified a CRC-associated region, 9q22.2-31.2, subsequently confirmed by analyses in CRC families [12–14]. Genes located within the region, such as *GALNT12*, *AXIN2* or *TGFBR1*, have been suggested as potential causal candidate genes [15–19]. Interestingly, our group could find support for the same locus in a linkage study in a large Swedish family (No. 24) with one individual affected with early onset rectal cancer and several relatives with adenomas (LOD = 2.4) [20]. A subsequent linkage study carried out by our group in 600 individuals from 121 non-FAP/non-LS families identified the exact same locus, as the second-best hit, although still not statistically significant (HLOD = 2.2) [4]. These results prompted further studies in an attempt to define the disease-causing mechanism within this locus in family No. 24 and other families that showed linkage to the same 9q region.

## RESULTS

After the first [20] and before the second [4] linkage study, all exons and exon-intron boundaries of all coding genes within the linked region were (Sanger) sequenced in two cancer/adenoma-affected members (Co-648 and Co-166) of Family No. 24 and no clear deleterious mutation was found (data now shown). All missense variants identified (Supplementary Table 1) were assessed by using association studies that included up to 400 CRC cases and controls, finding no clear association with the disease.

Allele-specific expression (ASE) of TGFBR1, located within the region of interest, was reported to be associated with an increased risk of CRC [19]. However, no TGFBR1 allelic expression imbalance was identified in RNA extracted from peripheral blood lymphocytes of the affected members of Family No. 24 (data not shown). Exon-targeted deletion/duplication analysis using a custom array-CGH design showed no pathogenic or likely pathogenic deletions or duplications in the 9q region in the two affected relatives of Family No. 24 (data not shown). Since the distance between the probes in the array was approximately 2.5Kb, the method was sensitive and accurate enough to capture any deletions or duplications larger than approximately 2.5Kb in the region.

After the second linkage study, whole-exome sequencing was carried out in two affected members of Family No. 24 (Co-166 and Co-213) and in 16 affected members from eight families (No. 8, 13, 275, 296, 350, 478, 740 and 918), which had contributed the most to the HLOD score >2 in the subset of 27 high-risk families studied [4].

Data from whole-exome sequencing for the region of interest were merged to one data set for analysis. After filtering, all exonic non-synonymous variants with a population MAF<20% (source: ExAC; http://exac.broadinstitute.org/) that were shared by the two affected relatives from Family No. 24 were selected. We next searched the other families for mutations in the selected genes. The only gene, which involved Family No. 24 and at least one other family, was *GRIN3A,* where 3 variants were identified: rs62000403, rs3739722 and rs10989563 (Supplementary Table 2). Genotyping of the three *GRIN3A* variants was carried out in 768 familial CRC cases and 768 controls. None of the three variants was associated with the disease (*p*-values: 0.3933, 0.1926 and 0.1840, respectively).

When looking for mutations in the other families only, one gene, *NUTM2G*, displayed variants in the 9q22 linked families. Three heterozygous *NUTM2G* missense variants were identified. Two families (275 and 296), carried rs201544487, one family (296) rs2296815 and a third family (918) rs7866127. All three variants were common in the European population (MAF range: 4.8–12.5%; source ExAC) and were predicted to be neutral by at least out of the *in silico* predictors used, suggesting a non-pathogenic nature (Supplementary Table 2).

The absence of suggestive deleterious mutations within the coding region included in the region of interest, led us to hypothesize that the region might hold a genetic risk factor within the non-exonic regions. Moreover, the presence of the 9q22 linkage in a total of Swedish families prompted us to test the hypothesis of a Swedish founder haplotype. Next, we performed a haplotype association study using 2664 consecutive CRC cases and 4782 controls from an ongoing GWAS (CORECT).

Genotypes for 500 markers in the region (rs16909975–rs12237372) were accessed and two windows (10 and 25) were studied, thus requiring a *p*-value lower than 0.00005 for statistical significance. One suggested risk haplotype was a 25-SNP haplotype with an OR of 1.8 and a *p*-value 0.000058 (borderline statistically significant) and a haplotype frequency of 2.7% in the normal population. To find out if known CRC families carried this haplotype, all 25 markers were genotyped in a separate set of 61 familial CRC cases and their relatives, to find out if any of those families could have the suggested haplotype. None of the 61 familial CRC haplotypes matched the 25 markers on the haplotype even when considering those not fully informative for all 25 SNPs. The cases in the association studies were consecutive cases, and 82% were sporadic. We hypothesized that perhaps the haplotype would be less prevalent among the familial cases to explain why we could not see this risk haplotype among our 61 familial cases. The results from single SNP analysis supported this hypothesis, since the SNP with the best *p*-value, rs6477733 (*p* = 0.00019, Supplementary Figure 1), 1Mb from the haplotype, was more prevalent in sporadic cases (3%) compared to familial (1%), suggesting a difference between familial and sporadic cases.

**Figure 1:**
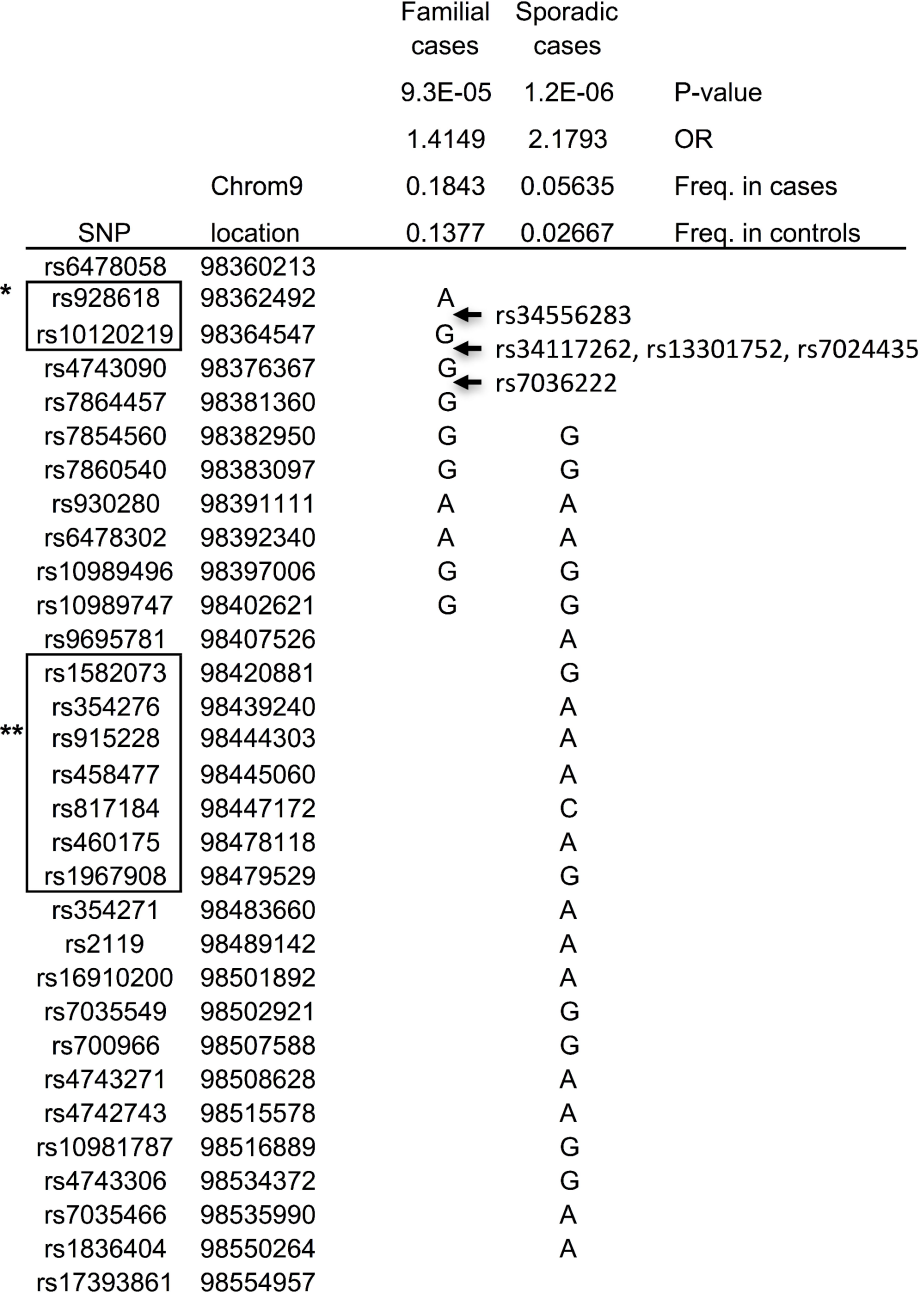
Haplotypes revealed in association studies ^*^RNA RP11-332M4.1; ^**^RNA RP11-180l4.2.

**Figure 2:**
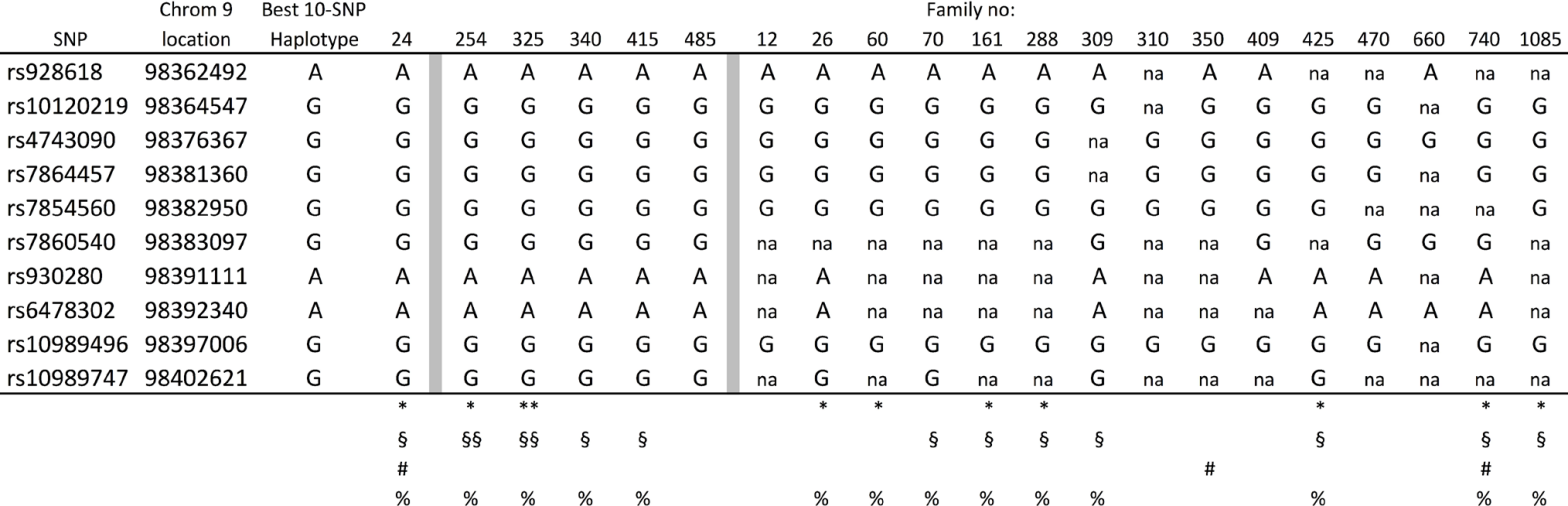
Families with the full or incomplete 10-SNP haplotype §,§§, heterozygous and homozygous for rs34117262, rs13301752, rs7024435, rs7036222; *,**, heterozygous and homozygous for rs34556283; na, not available; #, families linked to the region; %, families with targeted sequencing data.

To test this hypothesis, the 2664 CRC cases were split into 481 familial (those with at least one other CRC case in their family) and 2183 sporadic cases. The analysis was repeated, again using two windows (10 and 25), and this time familial cases and sporadic cases were analyzed separately, but using the same controls for both analysis. As a result, from the analysis of the sporadic cases the same 25-SNP haplotype was found with improved statistical significance (OR = 2.2, *P* = 0.0000012, haplotype frequency in normal controls 2.5%), confirming our hypothesis (Figure 1). The haplotype frequency of this 25-SNP haplotype in the 481 familial cases was estimated to be 1.8%, consistent with lack of this haplotype among the 61 familial cases. In the analysis using only familial cases, a different 10-SNP haplotype was suggested with an OR = 1.4 (*p* = 0.000093), and a haplotype frequency in normal controls of 13.7% (Figure 1). The frequency of this haplotype among sporadic cases was similar to controls, 14%, while the haplotype frequency in familial was 18%. The two haplotypes were overlapping for 6 markers (rs7854560, rs7860540, rs930280, rs6478302, rs109894996 and rs10989747). Since the 25-SNP haplotype was already tested and did not segregate within the separate 61 familial cases, we now tested the markers for the 10-SNP haplotype in the same 61 families, which included eight of the families from the linkage studies above. The full 10-SNP haplotype was found in one of the linked families, No. 24, and in five other families (254, 325, 340, 415, 485), as well as suggested (although not fully informative for all markers) in two of the other linked families (350, 740) plus 13 additional families (12, 26, 60, 70, 161, 288, 309, 310, 409, 425, 470, 660, 1085), in total 21 families (17%) corresponding well with the estimated haplotype frequency (18%) from the association study (Figure 2). These results confirm a 10-SNP-founder haplotype among Swedish familial cases and suggest a 25-SNP haplotype in Swedish sporadic CRC cases.

The region outlined by these two haplotypes, rs6478058-rs17393861, is in the intergenetic region between *PTCH1* and *LINC00046* (Figure 1). This region harbors two lincRNAs, *RP11-332M4.1* and *RP11-l80l4.2*. *RP11-332M4.1* is located within the non-overlapping part of the 10-SNP haplotype, while *RP11-l80l4.2* is located within the non-overlapping part of the 25-SNP haplotype (Figure 1).

Targeted sequencing of the whole region, suggested by our linkage study, was performed in samples from 46 families, including Family No. 24 and from families (254, 325, 340, 415) with the complete 10-SNP haplotype, and another 9 from 15 families with the suggested haplotype (26, 60, 70, 161, 288, 309, 425, 740, 1085). Since family No. 24 had most support for a genetic predisposition, based on both highest LOD and a fully informative 10-SNP-risk haplotype, two affected cousins (Co-213 and Co-166) from this family were selected for sequence analysis within the 10-SNP-risk haplotype (Supplementary Table 3). Since the association study suggested this haplotype to be present in 14% of the normal population, candidate variants with a population MAF<25% were selected for further analyses (Supplementary Table 3).

Nine variants were considered artifacts related to the difficulties for the annotation program to accurate interpret repeats. Two SNPs, rs3215956 and rs199596284, were ruled out as they showed the same frequency in 96 cases and 96 controls that were Sanger sequenced. Five variants in the two affected cousins in Family No. 24 remained as potential candidates in familial CRC cases. First, rs34556283, within *RP11-332M4.1*, with a population MAF of 17%, was also identified in two families with a complete haplotype (254 and 325), and in seven (26, 60, 161, 288, 425, 740, 1085) of the nine families with a suggested haplotype (Supplementary Table 3). Second, four SNPs, rs34227262, rs13301752, rs7024435 and rs7036222, (population MAF 19%) were located within the risk haplotype, but outside the lincRNA *RP11-332M4.1*. They were present in all four (254, 325, 340, 415) families with the complete haplotype, and in seven (70, 161, 288, 309, 425, 740, 1085) of the nine families suggested to have the haplotype (Supplementary Table 3). The rs34556283 variant was genotyped in 725 consecutive CRC cases and 671 controls, showing a difference between cases and controls with an OR similar to the OR from the haplotype analysis (18.5% in cases and 16.4% in controls; OR = 1.15; *p* = 0.2 n.s.). Testing one (rs7024435) of the four variants on the same haplotype, did not show any difference between cases and controls when genotyped in 320 cases and 341 controls (20.5% in cases and 20.7% in controls; OR = 0.99; *p* = 0.29 n.s.).

## DISCUSSION

The CRC candidate region on 9q22 has been suggested by several studies [12–14], although not in any previous CRC GWAS, which is surprising considering the relatively high OR (2.2) in sporadic cases in the present study. We think this might be explained by the fact that we did haplotype analysis rather than single SNP analysis. The best *p*-value in the single-SNP analysis was much less significant (0.00019) compared with our first haplotype analysis (0.000058). The results are consistent with what we found in our previous haplotype analysis [21], (Oncotarget, in press). Besides, this region holds also other known CRC genes, which could have influenced results from single-SNP GWAS [15–19].

The background for this study was the repeated findings suggesting a CRC susceptibility locus on chromosome 9q22. First, it was suggested by a sib-pair study [14], then was confirmed in familial CRC [13] and by us in a follow-up study in family No. 24 [20]. This family was one of the families contributing to a suggested locus in the 9q region already in an earlier linkage study in Finnish, Danish and Swedish families by Päivi Peltomäki (unpublished data), but when the sib-pair study was published, family No. 24 was extended to include more family members, and the published locus on 9q could be confirmed [20]. Still, no mutation was detected in the family using Sanger sequencing of all genes in the region (Bert Vogelstein, unpublished data).

The exact same locus came up again as a result in our recent linkage study of 126 families [4]. Thus, we decided to continue the search for genes in the region, now including these new families. Whole exome analysis in members of family No. 24 and other linked families did not find any support for a causative gene in the region. Since the suggestion for an increased risk came from both high-risk families [4, 13] and low/moderate risk families [14, 20], we decided to use an approach of haplotype analysis to search for a founder cause.

Data from an ongoing GWAS in CORECT, a consortium for association studies in CRC, was used to study this 9q region. The results suggested two separate risk factors with one haplotype each. When we analyzed the samples using sporadic and familial samples separately, we had support for our hypothesis of two founder effects. In support of the results, to 21 (all relatives were not fully informative for all 10 SNPs) of the CRC families included in the current study had the familial 10-SNP haplotype. None of the tested familial cases had the 25-SNP haplotype, suggested as risk factor in sporadic CRC, which was surprising but consistent with the low frequency among familial cases. The 25-SNP haplotype in the sporadic cases had an OR 2.2, while the 10-SNP haplotype in the familial cohort had an OR of 1.4. The relatively low OR in the familial cohort suggested a modifier role, probably exerting its effect together with other risk factors as expected in complex diseases, rather than as a high-risk gene. It was not possible to study haplotypes for sporadic cases, since no family members were collected in the Swedish Low Risk Study, which recruited consecutive CRC cases. Haplotypes could be studied in families, where both cases and relatives were recruited, when they were undergoing genetic counseling in. Most important, family No. 24, showed the full 10-SNP haplotype. Analysis of sequencing data for the region suggested one SNP, rs34556283, to possibly be the disease-causing variant within the RNA *RP11-332M4.1*.

The results suggested two risk loci, one in familial and one in sporadic CRC. Although it cannot be excluded that they both target the same risk locus, we think this is unlikely, since the support for the 25-SNP haplotype was stronger when the familial samples were removed. It is possible that both these loci, each with its own RNA, hold risk factors with a somewhat different effect on their own, or together with other genetic risk factors, and that the respective RNA is the target for the mutations. *RP11-332M4.1* and *RP11-l80l4.2* are long intergenic non-coding RNAs. They were manually annotated in the VEGA database [22] as part of the ENCODE project [23]. They are still poorly understood. LincRNAs has been suggested to be able to reprogram chromatin state as well as being involved in transcriptional silencing during cancer development [24–27]. The effect of mutations could relate to neighboring genes, such as the *PTCH1 or FANCC* gene or a processed transcript *LINC00046,* a protein coding gene *DKFZP434H0512* or *ERCC6L2*. The *PTCH1* gene is a well-known cancer gene involved in predisposition to basal cell carcinoma and other human tumors, but has also been implicated in CRC [28–30]. The *FANCC* gene is also well known to predispose to cancer and was recently also suggested in CRC [31]. *ERCC6L2* belongs to a family of helicases related to yeast *Snf2*, and mutations have been implicated in DNA repair and mitochondrial function [32]. A previous study also used a haplotype approach, in familial samples and could define two regions, both close but proximal to our region [12].

Even if Sweden today is not a very homogenous population, it was more so when the CRC patients were born, and our study demonstrates how novel risk factors can be found in such a population using haplotype analysis. It also demonstrates how linkage analysis not only can be used to find high-penetrant susceptibility loci, but also low-risk variants involved in complex disease. The difficulties to define a genetic variant outside the exome are obvious. Here, at least one variant was suggested, but it cannot be ruled out that limitations in current status of NGS have hidden other possible variants. Furthermore, it will be challenging to demonstrate the effect of a specific non-exonic variant.

We conclude that this study suggested two different risk alleles within the 9q22 locus. One, involving the RNA *RP11-l80l4.2,* was suggested in sporadic CRC (OR 2.2) and the other involving the RNA *RP11-332M4.1* in familial CRC (OR 1.4) suggesting the latter to act as a modifier or in complex inheritance with other genetic risk factors. Further studies will show how the risk alleles at this risk locus on 9q22 influence the risk of CRC.

## MATERIALS AND METHODS

### Swedish study participants

#### Familial cases used for sequencing- and haplotype analysis:

Familial cases were defined as coming from families where at least two first or second-degree relatives were affected with CRC. Family No. 24 was described in [20]. The families from the second linkage study were described in [4]. In total, whole-exome sequencing was performed in 98 familial CRC cases, which included family members from the families linked to the region. All CRC families were recruited through the Department of Clinical Genetics, Karolinska University Hospital Solna (Sweden). All families had undergone a full genetic investigation, and FAP and Lynch syndrome were excluded in all families using current clinical routines [33]. Two family members from each of a total of 61 CRC families were interrogated for the specific haplotypes. One case and one parent or child were genome-wide genotyped in order to analyze the 61 haplotypes to search for any candidate risk haplotype resulting from our studies.

#### CRC patients and controls used for association studies

The genotyping data used for the association haplotype study of the region, was obtained from CRC patients recruited in a nationwide study, the Swedish Low-risk Colorectal Cancer Study. The cases were from a cohort of more than 3300 consecutive CRC patients from 14 hospitals in and around Stockholm and Uppsala between 2004 and 2009, and gave informed consent and blood for genetic studies. All cases were interviewed, by the same person, about their family history of CRC and other malignancies. Cancer in first- and second-degree relatives and cousins was recorded, and pedigrees for the families of the index-person (the patient) were constructed. All diagnoses in family members, which could have been CRC were verified using medical records or death certificates. Other diagnoses were coded as stated by the index case. Cases with no relatives diagnosed with CRC were considered sporadic. Familial CRC was defined as cases with at least one relative with CRC in the family as defined above. All patients where relatives were at increased risk because of the family history were offered genetic counselling. Sex, age and tumor location of the index-patients were recorded based on medical records. Tumors were assigned locations in caecum, ascending colon, hepatic flexure, transverse colon, splenic flexure, descending colon, sigmoid colon or rectum. All tumors underwent evaluation directly after surgery by a local pathologist. The tumors were staged according to both AJCC classification and TNM system. From all patients in that study, detailed pedigrees were obtained to be able to classify each case as familial or, mostly, sporadic. As controls were used 4782 healthy unrelated twins from the Swedish Twin registry [34].

### Array-CGH

A custom designed array-CGH analysis was used for exon targeted detection of deletions and duplications in the 9q region. Agilent Technologies SureDesign was used to design the targeted 4x180K array (Oxford Gene Technologies, Oxfordshire, UK). This design has 8908 probes targeting the 9q region with a median probe spacing of 818 base-pairs giving a resolution using a 3probe cut-off of about 2.5 Kb. Experiments were performed at the Department of Clinical Genetics at Karolinska University Hospital, Stockholm, Sweden according to the manufacturer’s protocol. Slides were scanned using the Agilent Microarray Scanner (G2505C, Agilent technologies, USA). Raw data were normalized using Feature Extraction Software (10.7.3.1, Agilent Technologies, USA), and log2 ratios were calculated by dividing the normalized intensity in the sample by the mean intensity across the reference sample. The log2 ratios were plotted and segmented by circular binary segmentation in the CytoSure Interpret software (Oxford Gene Technology, Oxfordshire, UK). Oligonucleotide probe positions were annotated to the human genome assembly hg19 (www.genome.ucsc.edu).

### Sanger sequencing

Sanger sequencing was performed as previously described [35, 36]. Primer pairs were designed to amplify the coding regions of all genes in the 9q region. PCR products were purified using Agencourt AMPure Beads (Beckman Coulter) and sequenced with nested PCR primers. Sanger sequencing data was analyzed as previously [35].

### Exome sequencing of germline DNA from 98 familial CRC cases

DNA was quantified using a Qubit Flurometer (Life Technologies). Sequencing libraries were prepared according to the TruSeq DNA Sample Preparation Kit EUC 15005180 or EUC 15026489 (Illumina). Briefly, 1–1.5 ug of genomic DNA was fragmented using a Covaris (Covaris, Inc.). Thirty-seven of the DNA samples were fragmented according to the Covaris 400 bp protocol and 61 samples were fragmented according to the SureSelect Protocol. After fragmentation, all samples were subjected to end-repair, A-tailing, and adaptor ligation of Illumina Multiplexing PE adaptors. An additional gel-based size selection step was performed for the 37 samples. The adapter-ligated fragments were subsequently enriched by PCR followed by purification using Agencourt AMPure Beads (Beckman Coulter). Exome capture was performed by pre-pooling equimolar amounts and performing enrichment in 5- or 6-plex reactions according to the TruSeq Exome Enrichment Kit Protocol (EUC 15013230). Library size was checked on a Bioanalyzer High Sensitivity DNA chip (Agilent Technologies) while concentration was calculated by quantitative PCR. The pooled DNA libraries were clustered on a cBot instrument (Illumina) using the TruSeq PE Cluster Kit v3. Paired-end sequencing was performed for 100 cycles using a HiSeq 2000 instrument (Illumina) with TruSeq SBS Chemistry v3, according to the manufacturer’s protocol. Base calling was performed with RTA (1.12.4.2 or 1.13.48) and the resulting BCL files were filtered, de-multiplexed, and converted to FASTQ format using CASAVA 1.7 or 1.8 (Illumina). Data have been analyzed using the bcbb package (https://github.com/bbcb). After sequencing, the samples have been aligned to the reference genome hg19GRCh37 using BWA [37], sorted and PCR duplicates were removed with Picard (http://broadinstitute.github.io/picard/). The calculation of mapping and enrichment statistics were done with Picard and GATK. Variants were called using GATK and followed a best practice procedure implemented at the Broad Institute [38].

### Mutation annotation

The output mutations in variant call format (VCF) were annotated using ANNOVAR [39], which generated an excel-compatible file with gene annotation, amino acid change annotation, dbSNP identifiers [40], and 1000 Genomes Project allele frequencies [41].

### Genotyping and quality control of the association study

DNA was extracted from peripheral blood samples for both the cases and the controls. The 2690 cases were genotyped at the Center for Inherited Disease Research at Johns Hopkins University, US, using the Illumina Infinium^®^ OncoArray-500K BeadChips. The 4782 controls from the Swedish TwinGene registry were genotyped in Uppsala, Sweden using the Illumina OmniExpress BeadChips. The twin cohort and the Colorectal Cancer Transdisciplinary Study (CORECT) cohort went through quality control (QC) at their corresponding genotyping centers. In total 240370 SNPs were shared between the two platforms on which the data was merged and TOP strand format was accounted for. 9117 (2690 cases and 6427 controls) individuals were proceeded for QC analysis. In the first QC round (QC1), heterozygous haploid genotypes were excluded as well as samples with gender inconsistency and same position variants. The 239113 SNPs and 9114 individuals (2688 cases and 6426 controls) passed QC1. A second QC stage (QC 2) was performed on the merged data, where SNPs with <98% call rate, <1% minor allele frequency (MAF) and those inconsistent with Hardy–Weinberg (hwe 0.0001) equilibrium in controls were removed. 223065 SNPs remained after QC 2. In the third and final QC (QC 3) a multidimensional scaling (MDS) analysis was conducted on all the remaining markers for the purpose of population stratification and to identify ethnic outliers. The outliers were excluded from the dataset while the rest were plotted in an MDS plot (Supplementary Figure 1). After QC 3, 223065 SNPs and 9068 individuals (2664 cases, 6408 controls) remained to perform further downstream analyses.

### Genome-wide association study

Haplotype association studies were performed using PLINK V1.07 [42] on three sub-groups of CORECT genotyping data, familial (*n* = 481), sporadic (*n* = 2183), and familial + sporadic (*n* = 2664) as cases, and Swedish Twin Registry [34] as controls.

### Genotyping of familial samples for testing of haplotypes

Genomic DNA was extracted from peripheral blood using standard procedures. Genotyping of in total 587 individuals, familial CRC cases and their relatives, was performed using the Illumina HumanOmniExpress-12v1_H BeadChip. The results, 730,525 SNPs, were analyzed using the software GenomeStudio 2011.1 from Illumina Inc. Average sample call rate per SNP with sample call rate >0 was >99% and the overall reproducibility >99.99%. Arrays were processed according to manufactures protocol at the SNP&SEQ Technology Platform at Uppsala University and available on request (www.genotyping.se).

### Targeted sequencing of the 9q region

Capture sequencing of 46 familial CRC patients was performed by Axeq Technologies, US, using a SureSelect target enrichment system process followed by 100 bp paired-end sequencing on an Illumina HiSeq2000 sequencer. After sequencing, bioinformatics analysis of the FASTQ files included alignment of sequence reads to the reference human genome (GRCh37/hg19) using BWA and SAMTools, applying GATK [38, 43, 44] base quality score recalibration, indel realignment, duplicate removal, variant calling and annotation (dbSNP and 1000 Genome Project).

### Association studies of missense mutations

Association studies were performed using Taqman SNP Genotyping Assay (Thermo Fisher Scientific).

### Ethics

All patients gave written informed consents in accordance with Swedish legislation (2003:460) and the study was approved by the Regional Research Ethics Committee, Dnr: 2002-20489, 2008/125-2031/2, 2014/1324-31 and 2016/24-31/1.

## SUPPLEMENTARY MATERIALS FIGURE AND TABLES







## Abbreviations

SNP: single nucleotide polymorphism
LOD: logarithm of odds
HLOD: heterogeneity LOD
OR: odds ratio
MAF: minor allele frequency
Mb: megabase
