# Supplementary material for: Two novel colorectal cancer risk loci in the region on chromosome 9q22.32
Source: Oncotarget. 2018 Jan 29;9(13):11170–9. doi: 10.18632/oncotarget.24340 (PMC5834248; doi:10.18632/oncotarget.24340)
Supplement: Supplementary file 1 [file oncotarget-09-11170-s001.pdf]

Two novel colorectal cancer risk loci in the region on chromosome 9q22.32

SUPPLEMENTARY MATERIALS

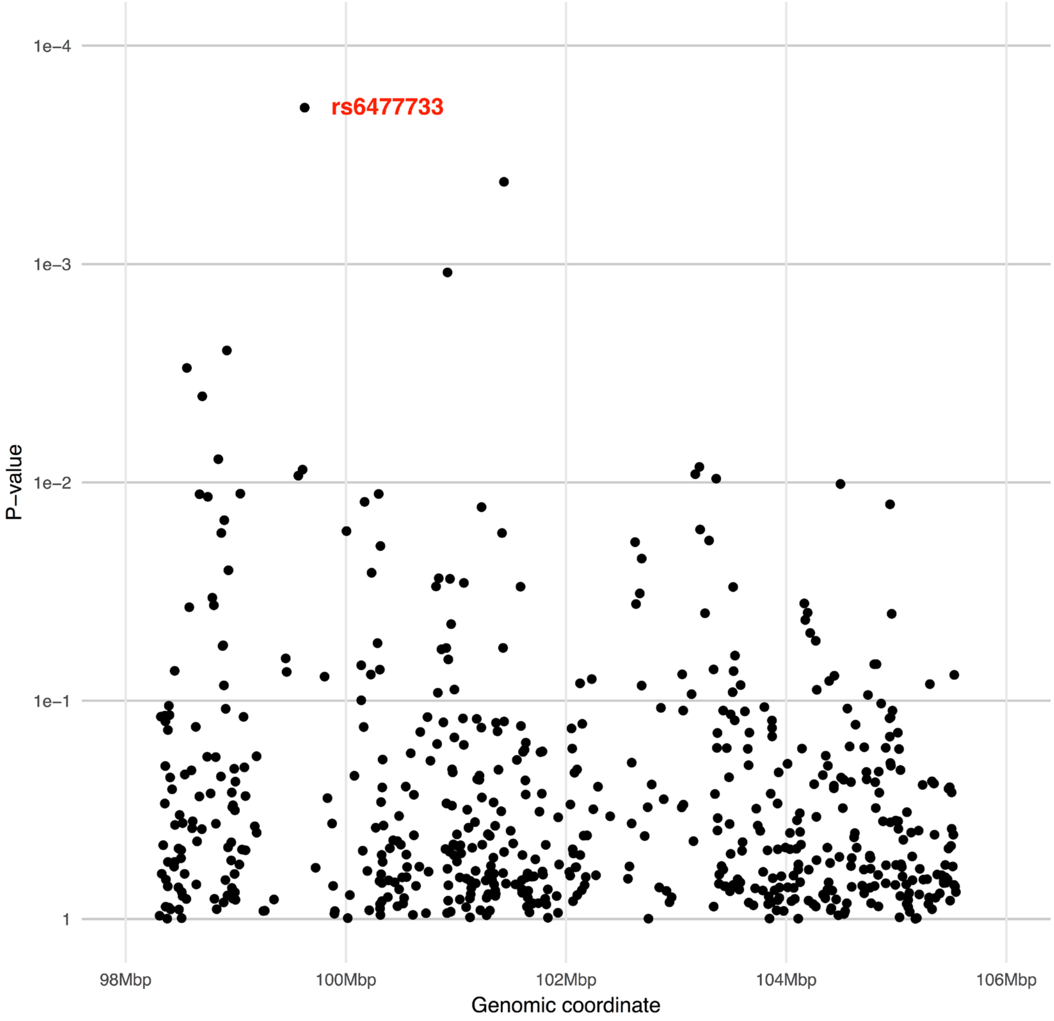

Supplementary Figure 1: SNP Association study in 9q region (all CORECT consecutive cases vs Swedish Twin controls).

Supplementary Table 1: Missense mutations from Sanger sequencing found in both samples

Gene name	hCT no	Exon	Type of exon	Co-166	Co-648
ZNF782	hCT1770260	Ex6	Last Exon	T/G	T/G
C9Orf97	hCT2273952	Ex3		T/T	T/G
CORO2A	hCT21111	Ex1	Start at nt 225	A/T	A/T
Predicted gene	hCT1653819	Ex4	Start at nt 79	C/T	C/T
GRIN3A	hCT20173	Ex2	Coding	G/G	G/G
		Ex9	Last Exon - 337	A/T	A/T

Supplementary Table 2: Missense mutations found in patients using exome sequencing. See Supplementary_Table_2

Supplementary Table 3: All variants (hom, het, oth) on the 10bp haplotype in two relatives from family 24. See Supplementary_Table_3
