# Supplementary material for: Two novel colorectal cancer risk loci in the region on chromosome 9q22.32
Source: Oncotarget. 2018 Jan 29;9(13):11170–9. doi: 10.18632/oncotarget.24340 (PMC5834248; doi:10.18632/oncotarget.24340)
Supplement: Supplementary file 2 [file oncotarget-09-11170-s002.docx]

**Supplemental Table 2. Missense mutations found in patients using exome sequencing**

| CHR | POS | REF | ALT | Func | Exonic Function | Gene | snp138 |  | minor allele frequency | | | In Silico predictor | | | | | | | |
| --- | --- | --- | --- | --- | --- | --- | --- | --- | --- | --- | --- | --- | --- | --- | --- | --- | --- | --- | --- |
|  |  |  |  |  |  |  |  | cytoBand | CRC cases | ExAC (ALL) | ExAC (NFE) | SIFT | Polyphen2 | LRT | MutationTaster | MutationAssessor | FATHMM | RadialSVM | LR |
| 9 | 99694397 | C | G | exonic | nonsynonymous_SNV | NUTM2G | rs2296815 | 9q22.33 | 0.0729 | 0.1308 | 0.0483 | Deleterious | Possibly damaging | Neutral | Polymorphism automatic | Neutral | Tolerated | Tolerated | Tolerated |
| 9 | 99699485 | G | C | exonic | nonsynonymous_SNV | NUTM2G | rs7866127 | 9q22.33 | 0.1562 | 0.1313 | 0.1187 | Deleterious | Probably damaging | Neutral | Polymorphism automatic | Medium | Tolerated | Tolerated | Tolerated |
| 9 | 99700710 | G | A | exonic | nonsynonymous_SNV | NUTM2G | rs201544487 | 9q22.33 | 0.0833 | 0.1005 | 0.1250 | Tolerated | . | Neutral | Polymorphism | Low | Tolerated | Tolerated | Tolerated |
| 9 | 104335586 | T | A | exonic | nonsynonymous_SNV | GRIN3A | rs62000403 | 9q31.1 | 0.1354 | 0.0532 | 0.0591 | Tolerated | Benign | Neutral | Disease causing | Low | Tolerated | Tolerated | Tolerated |
| 9 | 104335682 | C | T | exonic | nonsynonymous_SNV | GRIN3A | rs3739722 | 9q31.1 | 0.1146 | 0.1403 | 0.1174 | Tolerated | Benign | Neutral | Polymorphism automatic | Medium | Tolerated | Tolerated | Tolerated |
| 9 | 104385711 | C | T | exonic | nonsynonymous_SNV | GRIN3A | rs10989563 | 9q31.1 | 0.1875 | 0.2034 | 0.2443 | Tolerated | Probably damaging | Deleterious | Polymorphism automatic | Low | Tolerated | Tolerated | Tolerated |
