# Supplementary material for: Two novel colorectal cancer risk loci in the region on chromosome 9q22.32
Source: Oncotarget. 2018 Jan 29;9(13):11170–9. doi: 10.18632/oncotarget.24340 (PMC5834248; doi:10.18632/oncotarget.24340)
Supplement: Supplementary file 3 [file oncotarget-09-11170-s003.docx]

**Supplemental table 3. All variants (hom, het, oth) on the 10bp haplotype in two relatives from family 24**

| CHROM | POS | REF | ALT | Function | Neighboring genes | snp138 | 1000g2014oct_eur | 1000g2014oct_all | Frequency in our samples | cytoBand | 24 | 24 | 254 | 325 | 340 | 415 | 26 | 60 | 70 | 161 | 288 | 309 | 425 | 740 | 1085 |
| --- | --- | --- | --- | --- | --- | --- | --- | --- | --- | --- | --- | --- | --- | --- | --- | --- | --- | --- | --- | --- | --- | --- | --- | --- | --- |
|  |  |  |  |  |  |  |  |  |  |  | Co-166 | Co-213 | Co-616 | Co-851 | Co-831 | Co-1031 | Co-135 | Co-190 | Co-200 | Co-809 | Co-1141 | Co-783 | Co-1458 | Co-1375 | Co-1518 |
| 9 | 98362219 | CGGG | C | intergenic | PTCH1,LINC00476 | rs3215956 | 0.171 | 0.227236 | 0.2292 - 7-allele | 9q22.32 | het | oth | het | het | oth | wt | het | het | oth | oth | wt | oth | hom | het | het |
| 9 | 98362219 | CGGG | CGG | intergenic | PTCH1,LINC00476 | . |  |  | 0.2396 - 9-allele | 9q22.32 | het | het | oth | oth | oth | wt | oth | oth | het | het | wt | het | oth | oth | oth |
| 9 | 98362219 | CGGG | CG | intergenic | PTCH1,LINC00476 | . |  |  | 0.0417 - 8-allele | 9q22.32 | oth | oth | oth | oth | het | wt | oth | oth | oth | oth | wt | oth | oth | oth | oth |
| 9 | 98362219 | CGGG | CGGGG | intergenic | PTCH1,LINC00476 | . |  |  | 0.0104- 11-allele | 9q22.32 | oth | oth | oth | oth | oth | wt | oth | oth | oth | oth | wt | oth | oth | oth | oth |
| 9 | 98365343 | A | G | intergenic | PTCH1,LINC00476 | rs10985640 | 0.4334 | 0.625 | 0.3854 | 9q22.32 | hom | het | het | hom | het | wt | het | het | wt | het | het | het | het | het | het |
| 9 | 98366851 | G | A | intergenic | PTCH1,LINC00476 | rs10818898 | 0.3976 | 0.615016 | 0.2812 | 9q22.32 | het | het | het | hom | het | wt | het | het | wt | het | het | wt | het | het | het |
| 9 | 98367636 | G | A | intergenic | PTCH1,LINC00476 | rs34556283 | 0.171 | 0.223842 | 0.2083 | 9q22.32 | het | het | het | hom | wt | wt | het | het | wt | het | het | wt | het | het | het |
| 9 | 98367717 | C | T | intergenic | PTCH1,LINC00476 | rs10818991 | 0.4235 | 0.657548 | 0.3750 | 9q22.32 | hom | het | het | hom | het | wt | het | het | wt | het | het | wt | het | het | het |
| 9 | 98368761 | T | C | intergenic | PTCH1,LINC00476 | rs1984119 | 0.2654 | 0.236222 | 0.2917 | 9q22.32 | het | het | het | hom | wt | wt | het | het | wt | het | het | wt | het | het | het |
| 9 | 98371097 | C | G | intergenic | PTCH1,LINC00476 | rs6478146 | 0.2893 | 0.345847 | 0.3021 | 9q22.32 | het | het | hom | hom | het | het | het | het | het | hom | het | het | het | het | het |
| 9 | 98371810 | T | C | intergenic | PTCH1,LINC00476 | rs10760582 | 0.5696 | 0.641973 | 0.5000 | 9q22.32 | hom | het | hom | hom | hom | het | het | het | het | het | het | hom | het | hom | het |
| 9 | 98372204 | GTT | GTTT | intergenic | PTCH1,LINC00476 | . |  |  | 0.2708 | 9q22.32 | oth | het | oth | oth | oth | het | het | het | het | oth | het | het | oth | oth | het |
| 9 | 98372204 | GTT | G | intergenic | PTCH1,LINC00476 | . |  |  | 0.1354 | 9q22.32 | het | oth | het | het | het | het | oth | oth | het | oth | oth | oth | oth | het | oth |
| 9 | 98372204 | GTT | GT | intergenic | PTCH1,LINC00476 | . |  |  | 0.1875 | 9q22.32 | oth | oth | het | oth | oth | oth | het | het | oth | het | oth | oth | het | oth | het |
| 9 | 98372204 | GTT | GTTTTT | intergenic | PTCH1,LINC00476 | . |  |  | 0.0833 | 9q22.32 | het | oth | oth | oth | oth | oth | oth | oth | oth | oth | oth | oth | het | oth | oth |
| 9 | 98373350 | G | A | intergenic | PTCH1,LINC00476 | rs34117262 | 0.1998 | 0.207268 | 0.2396 | 9q22.32 | het | het | hom | hom | het | het | wt | wt | het | het | het | het | het | het | het |
| 9 | 98373591 | G | A | intergenic | PTCH1,LINC00476 | rs13301752 | 0.1998 | 0.213458 | 0.2396 | 9q22.32 | het | het | hom | hom | het | het | wt | wt | het | het | het | het | het | het | het |
| 9 | 98374031 | A | T | intergenic | PTCH1,LINC00476 | rs7024435 | 0.1998 | 0.209665 | 0.2396 | 9q22.32 | het | het | hom | hom | het | het | wt | wt | het | het | het | het | het | het | het |
| 9 | 98377896 | A | G | intergenic | PTCH1,LINC00476 | rs7041767 | 0.5656 | 0.637979 | 0.4896 | 9q22.32 | hom | het | hom | hom | hom | het | het | het | het | het | het | hom | het | hom | het |
| 9 | 98379525 | A | G | intergenic | PTCH1,LINC00476 | rs4743093 | 0.6163 | 0.816094 | 0.5104 | 9q22.32 | hom | het | hom | hom | hom | het | het | het | het | het | wt | hom | het | hom | het |
| 9 | 98380246 | T | C | intergenic | PTCH1,LINC00476 | rs7851863 | 0.6093 | 0.798522 | 0.5208 | 9q22.32 | hom | het | hom | hom | hom | het | het | het | het | het | het | hom | het | hom | het |
| 9 | 98380279 | G | A | intergenic | PTCH1,LINC00476 | rs72754365 | 0.2734 | 0.236422 | 0.2917 | 9q22.32 | het | het | hom | hom | het | het | het | het | het | het | het | het | het | het | het |
| 9 | 98380588 | G | A | intergenic | PTCH1,LINC00476 | rs7036222 | 0.1998 | 0.210264 | 0.2396 | 9q22.32 | het | het | hom | hom | het | het | wt | wt | het | het | het | het | het | het | het |
| 9 | 98382007 | A | C | intergenic | PTCH1,LINC00476 | rs4743104 | 0.5656 | 0.666334 | 0.4896 | 9q22.32 | hom | het | hom | hom | hom | het | het | het | het | het | het | hom | het | hom | het |
| 9 | 98383097 | A | G | intergenic | PTCH1,LINC00476 | rs7860540 | 0.5885 | 0.755192 | 0.5104 | 9q22.32 | hom | het | hom | hom | hom | het | het | het | het | het | het | hom | het | hom | het |
| 9 | 98386457 | A | T | intergenic | PTCH1,LINC00476 | rs10120618 | 0.4046 | 0.528754 | 0.3750 | 9q22.32 | hom | het | hom | hom | hom | het | het | het | het | het | het | het | het | hom | het |
| 9 | 98388092 | C | T | intergenic | PTCH1,LINC00476 | rs1889618 | 0.2763 | 0.248203 | 0.2917 | 9q22.32 | het | het | hom | hom | het | het | het | het | het | het | het | het | het | het | het |
| 9 | 98388836 | C | CG | intergenic | PTCH1,LINC00476 | rs5899262 | 0.5447 | 0.670327 | 0.4583 | 9q22.32 | hom | het | hom | hom | hom | het | hom | het | het | het | het | hom | het | hom | het |
| 9 | 98389463 | TA | T | intergenic | PTCH1,LINC00476 | . | 0.2455 | 0.27476 | 0.4479 | 9q22.32 | oth | het | het | het | het | het | het | het | het | het | het | het | het | het | het |
| 9 | 98389463 | TA | TAA | intergenic | PTCH1,LINC00476 | . |  |  | 0.0729 | 9q22.32 | het | oth | oth | oth | oth | oth | oth | oth | oth | oth | oth | oth | oth | oth | oth |
| 9 | 98390785 | G | C | intergenic | PTCH1,LINC00476 | rs1889617 | 0.5447 | 0.676717 | 0.4583 | 9q22.32 | hom | het | hom | hom | hom | het | hom | het | het | het | het | hom | het | hom | het |
| 9 | 98391111 | C | T | intergenic | PTCH1,LINC00476 | rs930280 | 0.4583 | 0.53754 | 0.4167 | 9q22.32 | het | het | hom | hom | het | het | hom | het | het | het | het | hom | het | het | het |
| 9 | 98391372 | T | C | intergenic | PTCH1,LINC00476 | rs6478289 | 0.5447 | 0.692692 | 0.3958 | 9q22.32 | hom | het | hom | hom | hom | het | hom | het | het | het | hom | hom | het | . | het |
| 9 | 98392182 | G | A | intergenic | PTCH1,LINC00476 | rs11793640 | 0.2813 | 0.238219 | 0.3021 | 9q22.32 | het | het | hom | hom | het | het | hom | het | het | het | het | het | het | het | het |
| 9 | 98392340 | G | T | intergenic | PTCH1,LINC00476 | rs6478302 | 0.3738 | 0.471446 | 0.3438 | 9q22.32 | hom | het | hom | hom | hom | het | hom | het | het | het | het | het | het | hom | het |
| 9 | 98393278 | T | G | intergenic | PTCH1,LINC00476 | rs7854580 | 0.3867 | 0.503195 | 0.3542 | 9q22.32 | hom | het | hom | hom | hom | het | hom | het | het | het | het | het | het | hom | het |
| 9 | 98393279 | T | C | intergenic | PTCH1,LINC00476 | rs60089480 | 0.3807 | 0.49361 | 0.3542 | 9q22.32 | hom | het | hom | hom | hom | het | hom | het | het | het | het | het | het | hom | het |
| 9 | 98393954 | G | T | intergenic | PTCH1,LINC00476 | rs16910073 | 0.3002 | 0.339657 | 0.3125 | 9q22.32 | het | het | hom | hom | het | het | hom | het | het | het | het | het | het | het | het |
| 9 | 98394336 | T | C | intergenic | PTCH1,LINC00476 | rs12346725 | 0.4493 | 0.46226 | 0.4167 | 9q22.32 | het | het | hom | hom | het | het | hom | het | het | het | het | hom | het | het | het |
| 9 | 98395085 | T | G | intergenic | PTCH1,LINC00476 | rs10819911 | 0.3797 | 0.486022 | 0.4062 | 9q22.32 | het | het | hom | hom | het | het | hom | het | het | het | het | het | het | het | het |
| 9 | 98395317 | T | C | intergenic | PTCH1,LINC00476 | rs375276 | 0.999 | 0.999601 | 1.0000 | 9q22.32 | hom | hom | hom | hom | hom | hom | hom | hom | hom | hom | hom | hom | hom | hom | hom |
| 9 | 98395318 | G | A | intergenic | PTCH1,LINC00476 | rs10819915 | 0.6024 | 0.740216 | 0.5417 | 9q22.32 | hom | het | hom | hom | hom | het | hom | het | het | het | het | hom | het | hom | het |
| 9 | 98395621 | A | G | intergenic | PTCH1,LINC00476 | rs4742700 | 0.6024 | 0.740016 | 0.5417 | 9q22.32 | hom | het | hom | hom | hom | het | hom | het | het | het | het | hom | het | hom | het |
| 9 | 98396168 | CAAAAAAAA | C | intergenic | PTCH1,LINC00476 | rs199596284 |  |  | 0.3333 | 9q22.32 | het | het | hom | hom | het | het | hom | het | het | het | het | het | het | het | het |
| 9 | 98396189 | A | G | intergenic | PTCH1,LINC00476 | rs12555329 | 0.3141 | 0.370407 | 0.3125 | 9q22.32 | het | het | hom | hom | het | het | hom | het | het | het | het | het | het | het | het |
| 9 | 98396639 | G | T | intergenic | PTCH1,LINC00476 | rs6478319 | 0.2942 | 0.256789 | 0.2708 | 9q22.32 | het | het | hom | hom | het | het | hom | het | wt | het | wt | het | het | het | het |
| 9 | 98396674 | T | C | intergenic | PTCH1,LINC00476 | rs7848462 | 0.4493 | 0.491214 | 0.4167 | 9q22.32 | het | het | hom | hom | het | het | hom | het | het | het | het | hom | het | het | het |
| 9 | 98397459 | T | C | intergenic | PTCH1,LINC00476 | rs4743120 | 0.6233 | 0.80651 | 0.5521 | 9q22.32 | hom | het | hom | hom | hom | het | hom | het | het | het | het | hom | het | hom | het |
| 9 | 98397997 | T | C | intergenic | PTCH1,LINC00476 | rs4743122 | 0.4702 | 0.527955 | 0.3854 | 9q22.32 | het | het | hom | hom | het | het | hom | het | wt | het | wt | hom | het | wt | het |
| 9 | 98400242 | T | C | intergenic | PTCH1,LINC00476 | rs4743123 | 0.6243 | 0.809105 | 0.5521 | 9q22.32 | hom | het | hom | hom | hom | het | hom | het | het | het | het | hom | het | hom | het |
| 9 | 98401512 | T | C | intergenic | PTCH1,LINC00476 | rs10989681 | 0.3817 | 0.42512 | 0.4062 | 9q22.32 | het | het | hom | hom | het | het | hom | het | het | het | het | het | het | het | het |
| 9 | 98402621 | A | G | intergenic | PTCH1,LINC00476 | rs10989747 | 0.2962 | 0.258387 | 0.3125 | 9q22.32 | het | het | hom | hom | het | het | hom | het | het | het | het | het | het | het | het |
| 9 | 98404241 | A | G | intergenic | PTCH1,LINC00476 | rs4742701 | 0.6243 | 0.809505 | 0.5521 | 9q22.32 | hom | het | hom | hom | hom | het | hom | het | het | het | het | hom | het | hom | het |
| 9 | 98406181 | C | T | intergenic | PTCH1,LINC00476 | rs7027192 | 0.2972 | 0.273762 | 0.3229 | 9q22.32 | het | het | hom | hom | het | het | hom | hom | het | het | het | het | het | het | het |
| 9 | 98407051 | T | C | intergenic | PTCH1,LINC00476 | rs10990083 | 0.5835 | 0.613419 | 0.5417 | 9q22.32 | hom | het | hom | hom | hom | het | hom | hom | het | het | het | hom | het | hom | het |
